# The assessment of quality of life and satisfaction with life of patients before and after surgery of an isolated apical defect using synthetic materials

**DOI:** 10.1186/s12894-020-00666-7

**Published:** 2020-07-20

**Authors:** Maciej Zalewski, Gabriela Kołodyńska, Anna Mucha, Łukasz Bełza, Krzysztof Nowak, Waldemar Andrzejewski

**Affiliations:** 1 Faculty of Health Sciences, Medical University of Wrocław, Wrocław, Poland; 2 Independent Public Health Care Center of the Ministry of the Interior and Administration in Wroclaw, Gynecology Department, Wroclaw, Poland; 3 Department of Physiotherapy, University School of Physical Education, Wrocław, Poland; 4 Department of Genetics, Wrocław University of Environmental and Life Sciences, Wroclaw, Poland; 5 Department of Physiotherapy, Opole Medical School, Opole, Poland

**Keywords:** Isolated apical defect, Quality of life, Surgical treatment

## Abstract

**Background:**

Pelvic floor static disorders constitute a significant clinical and social problem. The incidence of the problem increases with the age of female patients up to 80 years of age. Due to various methods of procedural treatment, eligibility for surgery should be carefully discussed with the patient. Ideally, the surgery should be effective and with the least possible number of complications. The objective of this study was to assess the quality of life of patients before and after the surgery of an isolated apical defect with the use of BSC mesh.

**Methods:**

The study involved 60 patients who were diagnosed with pelvic floor static disorder on the basis of physical examination. Standardised questionnaires were used to assess the quality of life and satisfaction with life: the Perceived Quality of Life (P-QOL) and the Satisfaction With Life Scale (SWLS).

**Results:**

The P-QOL results for each domain were higher in patients before surgery compared to the results obtained after the surgery. For almost all domains, the results obtained were statistically significant. The results obtained in the SWLS questionnaire in most answers also show that after the procedure there was an improvement in satisfaction with life in the examined female patients.

**Conclusion:**

In most patients, surgical treatment of an isolated apical defect using BSC mesh results in the subsidence of bothersome symptoms and improves the quality of life.

## Background

Pelvic organ prolapse (POP) constitutes a significant clinical and social problem. The symptoms worsen when intra-abdominal pressure increases, for instance during physical activity or coughing. The number of patients suffering from pelvic organ prolapse is not exactly known due to different definitions of the disorder and various systems of classification. According to the latest European data, the ailment concerns approximately 6–11% of women. American data suggest that the disorder is found in about 24% of women. The incidence of the problem increases with the age of female patients up to 80 years of age. In such cases, the need to perform surgery is determined in 20% of women [1–3].

Currently, the basic indication for surgical treatment is the occurrence of symptoms and ineffective conservative treatment. and the lack of consent of the patient for conservative treatment or ineffective conservative treatment. Surgery may be warranted if if there are symptoms associated with prolapse such as:

- strain and/or pain in the lower abdomen;
- urinary and/or faecal incontinence;
- recurrent bladder ailments (urinary urgency or pollakiuria);
- difficulty urinating and/or defecating;
- the need to change body position in order to urinate;
- limited sexual activity [4].

Due to various methods of procedural treatment, eligibility for surgery should be carefully discussed with the patient. Ideally, the surgery should be effective and with the least possible number of complications.

In 1990, A. Wattiez [5] performed the first laparoscopic sacrocolpopexy, which is a gold standard for treating pelvic floor static disorders [6]. The surgery gives very good treatment results in the absence of complications typical for vaginal mesh implants [7].

Therefore, new solutions have appeared, such as Noe’s [8] pectopexy (the suspension of the Cooper’s ligament) and the Dubuisson’s laparoscopic lateral suspension [9].

Nevertheless, it seems that in the case of older patients with a history of internal diseases, for whom laparoscopic or abdominal surgeries under general anaesthesia may pose too high risks, the only alternative is transvaginal surgery. Many vaginal approaches to apical suspension have been described [10–12]. Therefore, it is necessary to find a method which would combine low invasiveness of transvaginal surgery with high efficiency [13].

Pelvic organ prolapse significantly reduces the quality of life for women [14, 15]. The most common problems include incomplete bladder voiding, chronic urine retention, interrupted or impeded urination, as well as the need to support urination by engaging the abdominal prelum or the need to change body position in order to urinate [16, 17]. Nowadays, doctors not only focus on extending the life of their patients, but also on improving its quality. Contemporary medicine aims to improve the quality of life of the patient to the condition from before the disease. Therefore, researchers are increasingly interested in assessing quality of life of people affected by various diseases [18, 19].

The objective of this study was to assess the quality of life of patients before and after performing the surgery of an isolated apical defect using BSC synthetic mesh.

## Material and methods

### Design and data collection

The study involved 60 patients from the Gynaecology Department of the Hospital of the Ministry of Internal Affairs and Administration in Wroclaw, who were diagnosed with pelvic floor static disorders. The POP-Q scale and the modified classification of pelvic static disorders according to DeLancey were used to objectively assess the type and degree of the disorder (Table 1).

**Table 1:** Stages of POP-Q system measurement

**Stage 0**	**no prolapse is demonstrated**
**Stage 1**	the most distal portion of the prolapse is more than 1 cm above the level of the hymen
**Stage 2**	the most distal portion of the prolapse is 1 cm or less proximal or distal to the hymenal plane
**Stage 3**	the most distal portion of the prolapse protrudes more than 1 cm below the hymen but no farther than 2 cm less than the total vaginal length (for example, not all of the vagina has prolapsed).
**Stage 4**	vaginal eversion is essentially complete

[Persu C, Chapple CR, Cauni V. Pelvic Organ Prolapse Quantification System (POP–Q) – a new era in pelvic prolapse staging. J Med Life. 2011; 15 4(1): 79.]

All patients qualified for the study had an isolated apical defect POP-Q III C or POP-Q IV C. The vast majority were, however, patients with POP-Q III C. The aim of the study was to assess the quality of life in another less common group of patients. Before the beginning of the study, informed consent was obtained from all patients to participate in the study. All qualified patients completed the P-QOL and the SWLS questionnaires twice: before the surgery and 12 months after surgery. Patients were qualified for the study on the basis of specific inclusion criteria.

The inclusion criteria were the following:

- Patients with an isolated apical defect;
- Reproductive organ static disorder grade III or IV of the POP-Q scale;
- Level I defect on the modified DeLancey scale;
- Lack of consent to pessary treatment;
- Ineffective conservative treatment
- Age over 65.

All patients qualified for the project had a cervical or vaginal stump suspension procedure performed using the AMI BSC system. The BSC mesh is designed to induce neo-ligament formation by establishing a symmetrical, two-sided vaginal vault suspension from sacral cruciate ligament. The BSC mesh provides support that was previously provided by natural ligaments that no longer work [20]. In this case, the mesh is fixed with i-Stitch-sutured fixation directly to the sacrospinous ligament. The surgical technique used included:

- pre-operative treatment (estriol aplication - 4 weeks before surgery, antibiotic prophylaxis with a combination of cephalosporin and metronidazole aplication, vaginal disinfection, anus covering);
- incision in the posterior vaginal wall (3 cm distal to the vaginal apex);
- access to the sacrospinous ligament (a canal designated to admit the index finger of the surgeon is formed by advancing Metzenbaum scissors immediately under the vaginal wall horizontally in the direction of the pelvic side wall);
- dissection of a horizontal space under the cranial vaginal tissue (from the upper end of the longitudinal vaginal incision, the tissues of the rectovaginal septum are dissected off the posterior aspect of the vaginal wall);
- choosing the future fixation points for the BSC Mesh (it is a matter of personal preference, on which side the i-Stitch suture is placed first, the suture is advanced into the receiving groove, the sutures are not knotted but guided laterally to the thighs of the patient where they are held i.e. by short Kocher clamps);
- fixation of the tape to the underside of the vaginal apex or the posterior or anterior aspect of the cervix with 3 or 4 non absorbable polypropylene threads
- threading the sutures through the mesh (the prepositioned sutures are threaded through the mesh from posterior to anterior);
- Tyning of the sutures and cut short, bringing the mesh in contact with the patient’s tissue for the first time, the vaginal incision is closed with the pre-positioned running suture);
- Preparation for postoperative care (insertion of estriol ointment into the vagina, weekly prescribing vaginal estriol applications) [21].

### Measures

1. *The Perceived Quality of Life* (P-QOL) questionnaire is a generic instrument for assessing perceived QoL and the level of satisfaction with life. The questionnaire consists of 38 questions, of which the first twenty represent nine QoL domains (general health perception, prolapse impact, role limitations, physical limitations, social limitations, personal relationships, emotions, sleep/energy, and symptom severity measures). The next eighteen questions refer to the symptoms of pelvic organ prolapse. The responses range from ‘none/not at all’ through ‘slightly/a little’ and ‘moderately’ to ‘a lot’. A four-point scoring system for each item was used to measure the severity of the urogenital prolapse symptoms. No combined score is calculated for this questionnaire. If a woman has only one affected life domain and another woman has another affected domain, they are both considered symptomatic, though in different aspects of QoL [22, 23].

2. *The Satisfaction With Life Scale (SWLS)* questionnaire was developed by Diener et al., and a Polish adaptation was made by Juszczyński [24]. It is a tool used to analyse the satisfaction of the respondents with their current life. The questionnaire consists of 5 questions assessed on a 7-point scale. Values from 1 for “I strongly disagree” to 7 for “I strongly agree” were assigned to the answers to individual questions. The higher the score (min. 5 points, max. 35 points), the greater the feeling of satisfaction with life [25].

### Statistical analysis

The statistical analysis was carried out using the R programme. Descriptive statistics were applied to analyse the results obtained. The quality of life of patients from before the surgery and 12 months after the surgery was compared using the following tests: Wilcoxon matched-pairs test or the Fisher’s exact test. The analysis was carried out both for the results obtained from the P-QOL and the SWLS questionnaires. The value of *p* < 0.05 was considered to be statistically significant.

## Results

60 women with diagnosed POP-Q III C and IV C were qualified for the project. The average age of the patients was 70.28 (the range of 65–87 years old). Assessment was performed before surgery and 12 months after surgery using AMI BSC. The P-QOL results for each domain were higher (i.e. worse) in patients before the surgery compared to the results obtained after the surgery. For almost all domains, the results obtained were statistically significant (Table 2). Most of the answers given in the P-QOL questionnaire, which concerned urinary tract and bladder symptoms, were also statistically significant as the quality of life in their functioning after the surgery has significantly improved (Tables 3 and 4). After the surgery, statistically significant changes in other symptoms assessed in the P-QOL questionnaire were also observed (Table 5).

**Table 2:** Domain scores of the PQoL questionnaire from women before and after surgery

	**before surgery**	**after surgery**	***p*-value**
General Health *n* = 60	40 (0–80) 38.60 (17.67)	20 (0–80) 26.67 (17.86)	0.00002861
Prolapse Impact *n* = 60	50 (0–75) 38.60 (29.15)	0 (0–75) 18.86 (27.66)	0.000007581
Role Limitation *n* = 60	12.50 (0–75) 26.96 (30.60)	0 (0–75) 10.75 (23.79)	0.0001724
Physical Limitation *n* = 60	0 (0–75) 27.19 (30.26)	0 (0–75) 10.31 (21.54)	0.0001927
Social Limitation *n* = 60	0 (0–75) 19.30 (25.12)	0 (0–50) 7.02 (16.37)	0.0002904
Personal Relationship *n* = 60	0 (0–72.73) 17.53 (24.51)	0 (0–54.55) 7.31 (13.48)	0.01551
Emotion *n* = 60	0 (0–75) 26.17 (29.94)	0 (0–75) 12.72 (23.63)	0.00008613
Sleep Energy *n* = 60	25 (0–75) 24.12 (24.87)	0 (0–75) 13.60 (17.56)	0.001441
Severity Measures *n* = 60	13.33 (0–73.33) 19.42 (22.51)	0 (0–73.33) 9.47 (16.81)	0.0002301

**Table 3:** Urinary symptom responses from PQoL questionnaire from women before and after surgery

**urinary symptom**	**response**	**before surgery**	**after surgery**	***p*-value**
frequent visits to the toilet to urinate	does not occur not at all a little moderately a lot	6 6 10 31 7	25 11 9 11 4	0.0004818
sudden, very strong urge to urinate	does not occur not at all a little moderately a lot	7 3 16 22 12	27 4 15 10 4	0.001219
urinary incontinence associated with a strong need to urinate	does not occur not at all a little moderately a lot	14 2 15 13 16	31 2 10 10 7	0.01085
urinary incontinence associated with coughing	does not occur not at all a little moderately a lot	18 3 13 12 14	31 3 10 10 7	0.1474
weak urine flow	does not occur not at all a little moderately a lot	18 11 18 8 5	39 5 8 4 4	0.002271
strain when emptying the bladder	does not occur not at all a little moderately a lot	28 10 9 10 3	46 4 4 5 1	0.01501
dripping urine after emptying the bladder	does not occur not at all a little moderately a lot	23 9 12 12 4	38 3 7 10 2	0.05322

**Table 4:** Bowel symptom responses from PQoL questionnaire from women before and after surgery

**bowel symptom**	**response**	**before surgery**	**after surgery**	***p*-value**
feeling of incomplete bowel emptying after defecation	does not occur not at all a little moderately a lot	28 10 11 7 4	39 3 10 5 3	0.176
constipation, difficulty in defecation	does not occur not at all a little moderately a lot	32 10 6 5 7	41 6 3 6 4	0.4012
strain accompanying the defecation	does not occur not at all a little moderately a lot	31 13 6 4 6	41 9 3 3 4	0.4488
use of fingers to defecate	does not occur not at all a little moderately a lot	48 6 2 1 3	53 0 5 0 2	0.04262
frequency of defecation	more than once a day once a day once every two days once every three days once a week or less	8 39 6 4 3	4 42 8 4 2	0.7779

**Table 5:** Other symptom responses from PQoL questionnaire from women before and after surgery

**symptom**	**response**	**before surgery**	**after surgery**	***p*-value**
vaginal bulge disturbing in intercourse	does not occur not at all a little moderately a lot	29 16 4 7 4	48 7 3 0 2	0.001898
back pain occurring together with discomfort in the vagina	does not occur not at all a little moderately a lot	36 6 5 6 7	49 2 6 1 2	0.03176
vaginal discomfort	does not occur not at all a little moderately a lot	26 6 13 6 9	44 5 7 1 3	0.007846
bulge in a vagina	does not occur not at all a little moderately a lot	33 4 11 5 7	48 3 5 3 1	0.03191
heaviness or pressure in the vagina or downstomach at the end of the day	does not occur not at all a little moderately a lot	32 2 10 10 6	48 1 4 5 2	0.03223
thickening in the vagina making defecate difficult	does not occur not at all a little moderately a lot	38 7 11 0 4	51 3 3 2 1	0.01145

Data are presented as median (range) and mean (standard deviation). n denotes size of each group.

Given *p*-values are for the Wilcoxon test for dependent samples.

Data are presented as subgroups size. Given p-values are for the Fisher’s exact test.

Data are presented as subgroups size. Given p-values are for the Fisher’s exact test.

Data are presented as subgroups size. Given p-values are for the Fisher’s exact test.

The results obtained after performing the SWLS questionnaire indicate that after the surgery, patients’ satisfaction with life showed an upward trend (Table 6). The results did not obtain statistical significance, no less visible is the upward trend in the assessed aspect. Table 6 presents the results of individual questions in the questionnaire, including the number of people who provided the answer. No statistical significance was obtained for these data. Despite this, especially in questions number 3 and number 4 it is noticeable that after the surgery patients more often indicated that they were happier and could fulfill their life goals (Table 7).

**Table 6:** Domain scores of the SWLS questionnaire from women before and after surgery

	**before surgery**	**after surgery**	***p*-value**
total score n = 60	23.5 (5–35) 22.92 (6.58)	30 (8–35) 23.72 (6.81)	0.5044

**Table 7:** Responses from SWLS questionnaire from women before and after surgery

**question**	**response**	**before surgery**	**after surgery**	***p*-value**
In most aspects my life is close to my ideal	I definitely agree I agree I rather agree I neither agree nor I disagree I rather disagree I disagree I definitely disagree	7 8 19 11 3 6 6	8 11 14 13 3 4 7	0.9279
The conditions of my life are perfect	I definitely agree I agree I rather agree I neither agree nor I disagree I rather disagree I disagree I definitely disagree	9 13 13 11 7 5 2	11 12 15 11 5 5 1	0.9889
I am happy with my life	I definitely agree I agree I rather agree I neither agree nor I disagree I rather disagree I disagree I definitely disagree	8 15 18 11 2 2 4	9 19 18 5 3 2 4	0.8119
So far, I achieve important goals that I want in my life	I definitely agree I agree I rather agree I neither agree nor I disagree I rather disagree I disagree I definitely disagree	5 13 16 14 3 1 8	9 16 11 13 3 1 7	0.8794
If I could live my life again, I would change almost nothing	I definitely agree I agree I rather agree I neither agree nor I disagree I rather disagree I disagree I definitely disagree	11 14 10 5 3 11 6	12 13 6 6 9 6 8	0.4445

Data are presented as median (range) and mean (standard deviation). n denotes size of each group.

Given *p*-value is for the Wilcoxon test for dependent samples.

Data are presented as subgroups size. Given p-values are for the Fisher’s exact test.

## Discussion

The main aim of this study was to assess the quality of life in patients before and after the surgery of an isolated apical defect. The results showed that after the surgery the quality of life of the patients improved significantly, as in most cases the symptoms which significantly limited the daily functioning of the affected women disappeared. Recently, many studies assessing the quality of life of patients with isolated apical defect have been created. These studies are often multi-faceted and take place in different areas [26–32].

Rahkola-Soisalo et al. [33] also studied the role of the influence of the isolated apical defect surgery on the quality of life of the patients. 207 patients were qualified for their study, and they had an isolated apical defect procedure performed using the Vaginal Uphold™ system. 12 months after the procedure, they assessed the quality of life and sexual function of the patients using three standardised questionnaires. On the basis of the results obtained, the authors concluded that the quality of life of the patients improved significantly, while the sexual function deteriorated after the surgery.

Hüsch et al. [34] assessed the quality of life in patients after transvaginal pelvic floor static disorder surgery using mesh implants. The results obtained were compared with those of patients who did not show any symptoms of pelvic floor static disorder and who were in the same age group. A low complication rate as well as quality of life at a comparable level between patients undergoing the procedure and healthy women were found.

Similar conclusions were reached by Fünfgeld et al. [35], who assessed the efficacy of the procedure 12 and 36 months after performing the alloplastic mesh implantation. In their work, the authors focused more on functional assessment and anatomical repair of the defect. Quality of life assessment was an additional element.

Both this study and those cited above show the importance of assessing the quality of life in patients with pelvic floor static disorders. Effective surgical treatment not only eliminates the anatomical defect, but also improves the mental health of the patients. The results presented show that healthcare workers should assess and attempt to improve the quality of life of the patients. In addition to assessing the quality of life, an interesting issue may also be investigating the changes in the sex life of people undergoing POP surgery. This topic was not the subject of research in this article. Nevertheless, the authors recommend taking it in the future.

## Conclusion

1. Pelvic organ prolapse significantly reduces women’s quality of life.
2. Surgical treatment of an isolated apical defect using AMI BSC kit causes in most patients the regression of burdensome symptoms and improves their comfort of life.
3. The P-QOL questionnaire is a more useful tool for assessing the effect of treatment than the SWLS questionnaire and helps patients become aware of the improvement in their quality of life after the surgery.

## Data Availability

The datasets used and/or analyzed during the current study are available from the corresponding author on reasonable request.

## Abbreviations

BSC: Bilater Sacrospinous Colposuspension
POP-Q: Pelvic Organ Prolapse Quantification System
P-QOL: Perceived Quality of Life
SWLS: Satisfaction With Life Scale
WHOQOL: World Health Organisation Quality of Life
